# Evaluation of GenoType MTBDR*plus* for the detection of drug-resistant *Mycobacterium tuberculosis* on isolates from Karachi, Pakistan

**DOI:** 10.1371/journal.pone.0221485

**Published:** 2019-08-19

**Authors:** Sara Siddiqui, Meredith B. Brooks, Amyn A. Malik, Junaid Fuad, Ahsana Nazish, Safia Bano, Mercedes C. Becerra, Hamidah Hussain

**Affiliations:** 1 Global Health Directorate, Indus Health Network, Karachi, Sindh, Pakistan; 2 Department of Global Health and Social Medicine, Harvard Medical School, Boston, MA, United States of America; 3 Interactive Research and Development, Karachi, Sindh, Pakistan; 4 Department of Epidemiology, Emory University Rollins School of Public Health, Atlanta, Georgia, United States of America; 5 Indus Hospital–TB Laboratory, Indus Health Network, Karachi, Sindh, Pakistan; All India Institute of Medical Science - Bhopal, INDIA

## Abstract

**Objective:**

To compare the diagnostic performance of the GenoType MRBDR*plus* assay with the gold standard phenotypic drug susceptibility testing in the detection of drug resistance among culture isolates obtained from patients in Karachi, Pakistan.

**Design:**

*Mycobacterium tuberculosis* isolates were obtained from 96 consecutive tuberculosis patients found to have resistance to isoniazid from two health centers in Karachi (January-November 2017). Isolates were tested for drug resistance against rifampin and isoniazid using the MTBDR*plus* assay. Results were compared with conventional drug-susceptibility testing and the frequency of specific mutations were reported.

**Results:**

The MTBDR*plus* assay had a sensitivity for rifampin resistance of 98.8% (95% CI: 93.4–100) and for isoniazid resistance of 90.6% (95% CI: 83.0–95.6). The MTBDR*plus* assay showed mutations in *rpoB* in 81 of the 96 (84.4%) isolates. Of the 87 isolates showing resistance to isoniazid via the MTBDR*plus* assay, 71 (74.0%) isolates had mutations in the *kat*G gene only, 15 (15.6%) isolates had mutations in the *inh*A promoter region, and 1 (1.0%) showed mutations in both genes.

**Conclusion:**

The GenoType MTBDR*plus* assay in Pakistan can identify subgroups at high-risk of having isolates with mutations in the *kat*G and/or *inh*A genes. Understanding the local burden of these mutations have implications for local diagnostic and treatment guidelines.

## Introduction

For decades, tuberculosis (TB) has remained a significant public health threat, with 10.0 million new cases of TB and 1.6 million related deaths in 2017.[1] TB elimination efforts are complicated by the spread of drug-resistant (DR-) TB strains, which are more difficult to diagnose and treat. Global incidence of DR-TB is on the rise; in 2017 there were an estimated 558,000 new cases of rifampin-resistant (RR-) TB or multidrug-resistant (MDR-) TB, defined as resistance to at least two of the most powerful, first-line anti-TB drugs, rifampin (RIF) and isoniazid (INH).[1] Adequate diagnostic tools are essential to promptly identify the drug resistance pattern for each person sick with TB, subsequently informing appropriate treatment recommendations.

Conventional culture-based drug susceptibility testing (DST) is considered to be the gold standard for determining drug resistance, and is important to confirm the presence of resistance to tailor regimens to include drugs that are likely effective. However, phenotypic DST on solid medium takes months to yield final resistance results.[2] DST using liquid medium, such as the Bactec Mycobacteria Growth Indicator Tube (MGIT) 960 system (Becton Dickinson Diagnostic Systems, Sparks, MD) can yield resistance results more quickly. Molecular-based assays were designed to detect specific drug resistance-encoding mutations in *Mycobacterium tuberculosis* (*MTB*), and have the advantage of being able to achieve resistance results within 48 hours. One such test, the GenoType MTBDR*plus* (Hain Lifescience, Nehren, Germany) rapidly detects resistance to RIF and INH. The assay detects mutations in the *rpoB* gene for RIF resistance, in the *katG* gene, and in the *inhA* regulatory region gene.[3] In 2017, the World Health Organization endorsed the line probe assay (LPA) of MTBDR*plus* for the detection of RIF and INH resistance in acid-fast bacilli smear-positive sputum or MTB cultures.

INH has been one of the cornerstones of TB treatment since it was introduced in 1952 due to its strong bactericidal properties.[4] Resistance to INH has been associated with mutations in genes encoding the mycobacterial catalase-peroxidase (*kat*G), an enzyme that activates INH, and the InhA protein (*inh*A).[4,5] A mutation in the *kat*G region confers high-level INH resistance while a mutation in the *inh*A region confers low-level INH resistance.[6] Studies have shown that the presence of genetic mutations in *inh*A regulatory and coding regions can also lead to cross-resistance to ethionamide (Eto) due to the sharing of common pathways, whereas *MTB* isolates with a *kat*G mutation remain susceptible to Eto.[6,7] Thus, for individuals with isolates with an *inh*A mutation only, high-dose INH may be a viable treatment option, whereas when only a *kat*G mutation is present, Eto may be an adequate substitute. Understanding the underlying prevalence of *kat*G and *inh*A mutations, coupled with understanding the local incidence of TB and DR-TB, can better inform local programs to implement different diagnostic algorithms, to prescribe more effective treatments for TB disease or presumed sub-clinical TB infection, and to maintain appropriate drug stockpiles.

We aim to compare the diagnostic performance of the MRBDR*plus* assay with the gold standard phenotypic DST in the detection of drug resistance among culture isolates obtained from patients in Karachi, Pakistan, and to determine the local burden of *katG* and *inh*A mutations and their implication for local diagnostic and treatment guidelines.

## Materials and methods

The evaluation of GenoType MTBDR*plus* assays was conducted using TB patient isolates from two health facilities, the Indus Hospital (TIH) and Jinnah Postgraduate Medical Centre (JPMC), in Karachi, Pakistan between January and November 2017. JPMC is a public sector tertiary hospital managed and funded by the provincial government while TIH is a private, not-for-profit charity hospital. Both hospitals provide care for low-income populations residing within Karachi or surrounding rural areas. The TB clinics at both of these health facilities fall under the umbrella of the Programmatic Management of Drug-Resistant Tuberculosis team, led by the Indus Health Network. Patients reporting to both health facilities were routinely screened for TB and triaged to undergo appropriate diagnostic testing when appropriate.

Baseline sputum samples were collected from patients during their routine clinical TB evaluation and then transported to the TB laboratory at TIH for testing. Smear microscopy was performed, in which acid fast bacilli were stained using fluorescent stain. Samples were stored at two to eight degrees Celsius for one week prior to decontamination. The samples were decontaminated using N-acetyl-L-cysteine/NaOH method[8,9] with 4% NaOH and centrifuged. For the culture, the sample was inoculated onto solid Löwenstein-Jensen or liquid MGIT media, and identification was performed using the BACTEC NAP TB Differentiation Test Kit (Becton Dickinson, USA) and growth in acid–containing media. If the culture was positive on either medium, samples were then prepared for DST.

Isolates underwent conventional first- and second-line drug susceptibility testing (DST) using BACTEC MGIT 960 SIRE kits. Phenotypic DST was performed against rifampin (RIF), isoniazid (INH), streptomycin (S), ethambutol (EMB), pyrazinamide (PZA), amikacin (Am), kanamycin (Km), ethionamide (Eto), and ofloxacin (Ofx). The following critical concentrations of drugs were used: RIF 1.0 μg/mL, INH 0.1 μg/mL, S 1.0 μg/mL, EMB 5.0 μg/mL, PZA 100 μg/mL, Am 1.0 μg/mL, Km 2.0 μg/mL, Eto 5.0 μg/mL, and Ofx 2.0 μg/mL. Susceptibility was determined by comparison of growth on a control medium with growth on a drug-containing medium.

Samples that showed resistance to isoniazid on conventional DST were selected to undergo first-line LPA. A GenoLyse kit was used to extract DNA, amplification was then done using a thermal cycler, and then hybridization was carried out in a TwinCubator at 45°C. LPA was then performed through GenoType MTBDR*plus* v2 assay, according to the manufacturer’s instructions.[10] Samples were batch processed for convenience. Results were considered valid if conjugate control, TUB control, inhA locus control and rpoB locus control bands developed correctly as per manufacturer instructions. The absence of at least one of the wild-type bands or the presence of bands indicating a mutation in each drug resistance-related gene implied that the sample was resistant to the specific antibiotic. When the wild-type probes of a gene stained positive and there were no detectable mutations within the region examined, the sample was considered susceptible to the respective antibiotic.

For the purposes of this analysis, we retrospectively reviewed programmatic records to identify consecutive patients at the two study sites during the study timeframe who met the following criteria: (1) had first- and second-line phenotypic DST completed on baseline samples; (2) *MTB* isolates were resistant to INH on DST; and (3) individuals had a permanent residence within Karachi.

Demographic characteristics, including age and sex, were extracted from eligible patient’s clinical chart at each study site. Type of TB (pulmonary versus extra-pulmonary [EPTB]) and history of TB treatment are also recorded. We use history of prior TB treatment as a proxy for previous INH use, as local TB treatment guidelines recommend the use of INH unless there is known resistance to INH. We categorize resistance patterns based on phenotypic DST results as either INH mono-resistance, multidrug-resistant (MDR-) TB (resistant to INH and RIF), extensively-drug resistant (XDR-) TB (resistance to INH, RIF, Ofx, Amk, and Km), or poly-drug resistant (PDR-) TB (resistance to INH and any other drugs except RIF).

We report the frequency and percentage of all categorical data variables. Age is reported as mean and standard deviation (sd). We report characteristics of all study participants, as well as the drugs to which their isolates are resistant by phenotypic DST. Sensitivity, specificity, positive predictive value (PPV), and negative predictive value (NPV) are calculated based on the agreement between LPA compared to the gold standard DST culture. The precisions of the estimates are reported using 95% confidence intervals (CIs). We then describe the breakdown of *kat*G, *inh*A, and *rpoB* mutations amongst individuals with INH-resistant isolates by phenotypic DST, Eto-resistant isolates by phenotypic DST, and amongst individuals with prior TB disease.

All characteristics are reported by the total study population and statistical tests, including chi-squared, t-tests, or Fisher’s exact tests, were used to compare characteristics and frequency of drug resistance across the health facilities. All data were analyzed using SAS V9.4 (SAS Institute Inc., Cary, NC, USA).

### Ethics statement

This study was determined to be exempt by the Interactive Research & Development Institutional Review Board due to use of programmatic, de-identified data.

## Results

We identified 96 individuals for inclusion in the study, comprising of 70 (72.9%) individuals enrolled at TIH and 26 (27.1%) enrolled at JPMC. The mean age is 29.4 (sd: 13.6), 44 (45.8%) were male, 93 (96.9%) had pulmonary TB, and 58 (74.4%) had a history of TB. More individuals at TIH had a history of TB disease as compared to JPMC (45 [83.3%] versus 13 [54.2%]; p-value: 0.0007). All other patient characteristics were similar across hospitals.

### Phenotypic DST results

Table 1 summarizes the drug susceptibility patterns of isolates included in the study. All 96 individuals included had isolates resistant to INH by culture, while 82 (85.4%) had isolates resistant to RIF, 29 (30.2%) to PZA, 25 (26.0%) to Ofx, 27 (17.1%) to Eto, 15 (15.6%) to ETH, and less than 10.0% each to S, Amk, and Km. More individuals at JPMC were found to have rifampicin resistance as compared to TIH (26 [100.0%] versus 56 [80.0%]; p-value: 0.010). Resistance for all other drugs were similar across hospitals. Overall, resistance testing identified 77 (80.2%) individuals with MDR-TB, 10 (10.4%) with INH mono-resistance, and 5 (5.2%) with XDR-TB.

**Table 1:** Phenotypic DST profiles of 96 isolates.

Drug resistance	Total (n = 96)
**Individual drug resistance (via DST culture methods) **	
Isoniazid	96 (100.0%)
Rifampicin	82 (85.4%)
Pyrazinamide	29 (30.2%)
Ethambutol	15 (15.6%)
Ethionamide	17 (17.1%)
Streptomycin	8 (8.3%)
Ofloxacin	25 (26.0%)
Amikacin	8 (8.3%)
Kanamycin	5 (5.2%)
**Resistance pattern **	
Isoniazid monoresistant TB	10 (10.4%)
Poly-drug resistant TB	4 (4.2%)
Multidrug-resistant TB	77 (80.2%)
Extensively drug-resistant TB	5 (5.2%)

**Abbreviations:** DST = drug-susceptibility testing; TB = tuberculosis.

### Performance of GenoType MTBDR*plus* assay

Table 2 summarizes the performance of GenoType MTBDR*plus*. The sensitivity for RIF resistance was 98.8% (95% CI: 93.4–100), specificity was 92.9% (95% CI: 66.1–99.8), PPV was 98.8% (95% CI: 92.5–99.8), and NVP was 92.9% (95% CI: 64.8–98.9). The sensitivity for INH resistance was 90.6% (95% CI: 83.0–95.6). Specificity, PPV, and NVP cannot be calculated for INH resistance because all patients included in the study had an isolate resistant to INH by phenotypic DST. A false-negative result was identified in 9 (9.4%) isolates, in which LPA found the isolate to be susceptible to INH but resistant via phenotypic DST. Of these 9 isolates, 1 (11.1%) was also resistant to Eto by phenotypic DST.

**Table 2:** Performance of GenoType MTBDR*plus* assay compared to phenotypic DST.

Genotypic DST results (n = 96)	Phenotypic DST results		% Sensitivity	% Specificity	% PPV	% NVP
	Resistant	Sensitive	(95% CI)	(95% CI)	(95% CI)	(95% CI)
**RIF**						
**Resistant**	81	1	98.8 (93.4–100)	92.9 (66.1–99.8)	98.8 (92.5–99.8)	92.9 (64.8–98.9)
**Sensitive**	1	13				
**INH**						
**Resistant**	87	0	90.6 (83.0–95.6)	N/A	N/A	N/A
**Sensitive**	9	0				

**Abbreviations:** CI = confidence interval; DST = drug-susceptibility testing; INH = isoniazid; NPV = negative predictive value; PPV = positive predictive value; RIF = (rifampicin).

### Detection of mutations associated with drug resistance using MTBDR*plus*

Mutations in *rpoB* conferring resistance to RIF were detected in 81 of the 96 (84.4%) isolates. The RIF-resistant isolates displayed different mutations; the most frequently observed mutation was observed in 45 (55.6%) of isolates in the S531L position.

Of the 87 individuals who were found to have INH-resistant isolates by LPA, 71 (81.6%) had mutations in the *kat*G gene only, 15 (17.2%) had mutations in the *inh*A promoter region, and 1 (1.1%) showed mutations at both genes. The majority (68, 78.2%) of isolates had a mutation in the *kat*G gene with an amino acid change of S315T1, indicating high-level INH resistance, and 13 (14.9%) had a mutation in *inhA* gene, C15T, indicating low-level INH resistance. There were no significant differences in genetic mutations identified across the two study sites.

Table 3 summarizes the distribution of mutations identified by MTBDR*plus*.

**Table 3:** Drug resistance patterns using GenoType MTBDR*plus* assay.

**Drug resistance patterns**				**Mutations detected**	**# (%) of strains**
**RIF resistance pattern (*rpoB* gene) (n = 81)**					
**WT probes**		**Mutant probes**			
WT		MUT3		Unknown	2 (2.5%)
WTΔ1		-		F505L,T508A,S509T	1 (1.2%)
WTΔ1, WTΔ2		-		F505L,T508A,S509T,L511P	1 (1.2%)
WTΔ2		-		L511P	4 (4.9%)
WTΔ2, WTΔ3		-		Q513L,Q513P,del514-516	2 (2.5%)
WTΔ2, WTΔ3		MUT2A		Unknown	1 (1.2%)
WTΔ2, WTΔ7		MUT2A		L511P,H526Y	1 (1.2%)
WTΔ3		-		Unknown	1 (1.2%)
WTΔ3, WTΔ4		MUT1		D516V	2 (2.5%)
WTΔ3, WTΔ4		-		D516Y,del515	3 (3.7%)
WTΔ4, WTΔ5		-		del518,N518I	1 (1.2%)
WTΔ7		-		H526R, H526P, H526Q, H526N, H526L, H526S, H526C	4 (4.9%)
WTΔ7		MUT2A		H526Y	5 (6.2%)
WTΔ7		MUT2B		H526D	2 (2.5%)
WTΔ8		-		S531Q, S531W, L533P	6 (7.4%)
WTΔ8		MUT3		S531L	45 (55.6%)
**INH resistance pattern (n = 87)**					
***katG***		***inhA***		**Mutations detected**	**# (%) of strains**
WT probes	Mutant probes	WT probes	Mutant probes		
WT	MUT1	-	-	Unknown	1 (1.1%)
WTΔ	MUT1	WTΔ1	MUT1	S315T1,C15T	1 (1.1%)
WTΔ	-	-	-	Unknown	2 (2.3%)
WTΔ	MUT1	-	-	S315T1	68 (78.2%)
-	-	WTΔ1	MUT1	C15T	13 (14.9%)
-	-	WTΔ1	-	Unknown	1 (1.1%)
-	-	WTΔ2	MUT3A	T8C	1 (1.1%)

**Abbreviations:** INH = isoniazid; MUT = mutation; RIF = rifampin; WT = wild-type pattern with all respective bands visible; WTΔ = lack of hybridization to the wild-type probe.

Of the 17 individuals with INH- and Eto-resistant isolates on phenotypic DST, 16 (94.1%) were resistant to INH by LPA. Of these, 10 (62.5%) had mutations in the *kat*G gene while 5 (31.3%) had mutations in the *inh*A promoter region. One (6.3%) showed mutations at both *kat*G and *inh*A genes. Of the 58 individuals who reported a history of prior TB, indicating prior INH use, and had INH-resistant isolates on phenotypic DST, 54 (93.1%) were resistant to INH by LPA, as indicated by 48 (88.9%) having mutations in the k*at*G gene and 6 (11.1%) having mutations in the *inh*A promoter region. Individuals with Eto-resistant isolates and individuals with prior history of TB disease had similar breakdowns of mutations as the larger population, 10 (62.5%) and 46 (85.2%), respectively, having an amino acid change of S315T1, and 4 (25.0%) and 5 (9.3%), respectively, having a mutation in *inh*A gene, C15T.

Table 4 summarizes the distribution of mutations identified by LPA for all 16 individuals with Eto-resistant isolates on phenotypic DST and INH-resistant isolates by LPA, and all 54 individuals with a history of TB disease and INH-resistant isolates by LPA.

**Table 4:** Drug resistance patterns using LPA in individuals with ETO-resistance and prior TB history.

**Individuals with Eto-resistant isolates (n = 16)**				**Mutations detected**	**# (%) of strains**
**INH resistance pattern**					
***katG***		***inhA***			
WT probes	Mutant probes	WT probes	Mutant probes		
WTΔ	MUT1	-	-	S315T1	10 (62.5%)
WTΔ	MUT1	WTΔ1	MUT1	S315T1,C15T	1 (6.3%)
-	-	WTΔ1	MUT1	C15T	4 (25.0%)
-	-	WTΔ2	MUT3A	T8C	1 (6.3%)
**Individuals with prior history of TB disease (n = 54)**					
**INH resistance pattern**				**Mutations detected**	**# (%) of strains**
***katG***		***inhA***			
WT probes	Mutant probes	WT probes	Mutant probes		
WT	MUT1	-	-	Unknown	1 (1.9%)
WTΔ	MUT1	-	-	S315T1	46 (85.2%)
WTΔ	-	-	-	Unknown	1 (1.9%)
-	-	WTΔ1	MUT1	C15T	5 (9.3%)
-	-	WTΔ1	-	Unknown	1 (1.9%)

**Abbreviations:** Eto = Ethionamide; INH = isoniazid; MUT = mutation; RIF = rifampin; WT = wild-type pattern with all respective bands visible; WTΔ = lack of hybridization to the wild-type probe.

## Discussion

High-level INH resistance, as indicated by the presence of *kat*G mutations[11], was identified in three-quarters of a cohort of patients with isolates resistant to INH; low-level INH resistance, as indicated by *inh*A mutations, was identified in one-sixth of this cohort. Only one percent had mutations at both genes. Patients with a prior history of TB, which suggests previous INH exposure, displayed an even higher level of *kat*G mutations and lower level of *inh*A mutations. Our study demonstrated a higher sensitivity for detecting RIF and INH resistance via LPA as compared to that reported in another study conducted in Pakistan (RIF: 98.8% vs 79.2%; INH: 90.6% vs 71.7%).[12]

Numerous prevalence studies of *kat*G and *inh*A mutations have been conducted globally.[13] Our findings fall in the middle of the previously reported ranges globally for mutations at each gene, and on the lowest end of the range for having mutations in both genes present. The reports of the percentage of mutations in the *kat*G gene range from 31.8%[13] to 96.9%[14] of isolates tested, while the percentage of mutations in the *inh*A promoter region ranges from 1.5% to 45.9%.[13] The percent of having mutations at both genes ranges from 1.2%[12] to 23.0%.[15] For example, in comparing the percentage of each mutation to those found in similar settings, those identified in Karachi were lower than the 83.0% of *kat*G mutations[16] and the 21.4% of *inhA* mutations[6] found in individuals with isolates resistant to INH in India. Among individuals with both INH- and Eto-resistant isolates, the percentage of *inh*A mutation was double that of the overall population being studied. This coincides with knowledge that genetic mutations in *inh*A can result in cross-resistance to Eto.

Our study found that the majority of individuals found to be resistant to INH on LPA (79%) had a S315T alternation in the *kat*G gene, which is between two reports from other studies in Pakistan, where 97%[12] and 76%[17] had the S315T alteration. The C-15T mutation in the *inh*A promoter region was higher in our study (16%) than that reported by the two Pakistan-based studies, 8%[12] and 4%.[17]

Identifying the prevalence of *kat*G and *inh*A mutations gives insight into potential treatment options that can be used for individuals with DR-TB disease and presumed sub-clinical infection. In individuals with only high-level INH resistance (*kat*G mutations), high-dose INH cannot be used, leaving Eto as a potential alternative drug to prescribe. Of the patients who tested positive for *katG* mutations, 70% had previous history of TB and have likely been exposed to INH previously. Thus, prior INH use may indicate that an alternative drug choice should be considered, even in the absence of LPA testing. In individuals with low-level INH resistance only *(inh*A mutations), the minimum inhibitory concentration may be low enough to be exceeded by high-dose INH,[18] making it a potential drug substitution for these patients. A study conducted in Kanpur, India, demonstrated that study participants who were administered high-dose INH as an adjuvant to MDR-TB therapy showed better bacteriological treatment response, without an increase in toxicity.[19] Although *inh*A mutations confer Eto resistance[4], there are low levels of the mutation detected in the study population, indicating that it can still be considered as a potential therapeutic for DR-TB treatment or presumed sub-clinical TB infection treatment in this setting.

Understanding the relationship between these mutations and previous INH exposure can also inform diagnostic algorithms. Thus, by identifying subgroups who may be at highest risk of having these mutations, knowledge may be gained about the most effective treatment regimen to recommend and who should receive priority LPA diagnostic testing after confirmation of INH-resistance via conventional culture methods. The high sensitivity of the LPA test suggests that it could potentially be used as a point-of-care rapid test to identify resistance, associated mutations, and to inform appropriate treatment. Other studies, such as one conducted in Maharashtra, India, further demonstrate the accuracy and quickness of LPA as a diagnostic tool, with the added advantage of its ability to detection mutations.[20] The sensitivity of Genotype MTBDR*plus* was much higher in our cohort for both INH and RIF than identified elsewhere in Pakistan (90.6% vs 71.7% for INH; 98.8% vs 79.2% for RIF).[12]

There are several limitations to this study, including our relatively small cohort of only 96 individuals with isolates resistant to INH. We anticipate, however, that these results are representative of the larger population in the hospitals’ catchment areas due to the non-restrictive inclusion criteria and the heterogeneity of individuals served by the two study sites. Additionally, our study only included individuals with isolates resistant to INH on culture so we were unable to calculate the false-positive rate of detecting INH-resistance. This should be further studied prior to programmatic implementation because, due to the open-tube format of the LPA, cross-contamination may lead to increased false-positive results. Additionally, LPA can only detect a small proportion of mutations in INH resistance, rendering LPA alone to not be sufficiently accurate in predicting the level of isoniazid resistance for a single mutation in *kat*G or *inh*A. It is, of course, still more informative than only performing phenotypic DST.

In sum, as rapid molecular tests gain increased use to identify resistance patterns among patients sick with TB, more treatment algorithms will need to be defined to guide the most effective treatment options for individuals with specific mutations. Increased use of LPA will inform local programs about INH resistance levels, depending on the mutation detected, and ultimately provide insight as to which TB drugs (high-dose INH or Eto) may be appropriate and most effective in specific subgroups. Understanding local burdens of these mutations will aid providers to prescribe appropriate TB treatment, and programs to both appropriately define diagnostic and treatment algorithms, and to stockpile drugs based on the anticipated proportion of resistance and associated mutations in the population.
